# Place of Death in Patients with Lung Cancer: A Retrospective Cohort Study from 2004-2013

**DOI:** 10.1371/journal.pone.0161399

**Published:** 2016-08-23

**Authors:** Emma L. O’Dowd, Tricia M. McKeever, David R. Baldwin, Richard B. Hubbard

**Affiliations:** 1 Division of Public Health and Epidemiology, Clinical Sciences Building, Nottingham City Campus, Hucknall Road, Nottingham, United Kingdom; 2 Department of Respiratory Medicine, David Evans Building, Nottingham City Hospital, Hucknall Road, Nottingham, United Kingdom; Institut national de la recherche scientifique, CANADA

## Abstract

**Introduction:**

Many patients with cancer die in an acute hospital bed, which has been frequently identified as the least preferred location, with psychological and financial implications. This study looks at place and cause of death in patients with lung cancer and identifies which factors are associated with dying in an acute hospital bed versus at home.

**Methods and Findings:**

We used the National Lung Cancer Audit linked to Hospital Episode Statistics and Office for National Statistics data to determine cause and place of death in those with lung cancer; both overall and by cancer Network. We used multivariate logistic regression to compare features of those who died in an acute hospital versus those who died at home.

**Results:**

Of 143627 patients identified 40% (57678) died in an acute hospital, 29% (41957) died at home and 17% (24108) died in a hospice. Individual factors associated with death in an acute hospital bed compared to home were male sex, increasing age, poor performance status, social deprivation and diagnosis via an emergency route. There was marked variation between cancer Networks in place of death. The proportion of patients dying in an acute hospital ranged from 28% to 48%, with variation most notable in provision of hospice care (9% versus 33%). Cause of death in the majority was lung cancer (86%), with other malignancies, chronic obstructive pulmonary disease (COPD) and ischaemic heart disease (IHD) comprising 9% collectively.

**Conclusions:**

A substantial proportion of patients with lung cancer die in acute hospital beds and this is more likely with increasing age, male sex, social deprivation and in those with poor performance status. There is marked variation between Networks, suggesting a need to improve end-of-life planning in those at greatest risk, and to review the allocation of resources to provide more hospice beds, enhanced community support and ensure equal access.

## Introduction

Many patients with cancer die in an acute hospital bed although this may be the location they prefer the least.[1] Those who die in acute hospital beds have been shown to have a worse quality of life compared to those who die in hospices or at home.[2] In addition their caregivers are at increased risk of psychiatric problems after the bereavement.[2,3] There are also cost implications; research has shown that end of life care in hospital costs three times more than that delivered in the community.[4,5] The National Health Service (NHS) implemented the National End of Life Care Programme in 2004 with the aim of allowing more patients to receive end of life care in their preferred location.

Following this, Gao and colleagues looked at changes in place of death for all cancer deaths in England between 1993 and 2010.[6] Their work showed that the proportion of patients dying at home or in hospices in many cancer types has increased but in lung cancer, the biggest cause of cancer death worldwide, this does not seem to be the case.[6] Many patients with lung cancer are diagnosed at an advanced stage; in fact 30% of patients are dead within 90 days of diagnosis, however work suggests that clinicians may leave discussions about end-of-life care too late to allow patients to achieve their preferred place of death. [7,8]

These data suggest that there is a need to look at the lung cancer population in particular to try to understand whether there are any features specific to this cancer type which confers an increased risk of dying in an acute hospital setting. This study aimed to determine place and cause of death in patients with lung cancer; to identify which features are associated with dying in an acute hospital bed versus at home; to assess geographical variation in place of death in England and in addition to identify if time of death following diagnosis impacts on ability to achieve preferred place of death. A greater understanding of these factors may allow clinicians to provide better advanced care planning in those at greatest risk.

## Methods

### Patient population and data source

The National Lung Cancer Audit (NLCA) is a prospective database that collates information provided by 157 English NHS hospitals on demographic, tumour and treatment for patients with primary lung cancer. It is linked to data from Hospital Episode Statistics (HES), used to derive co-morbidity information from coded inpatient episodes, alongside information regarding any interventions and procedures performed, and Office of National Statistics (ONS) that provides information on date, place and cause of death. Data were provided to the researchers under the terms of a data sharing agreement from the Health and Social Care Information Centre (HSCIC). HES and ONS records were linked by staff at the HSCIC to the NLCA using NHS numbers, with a second check to ensure that dates of birth, post codes and sex match. These data were then pseudonomised to ensure that the researchers did not have access to any identifiable fields.

We identified all patients diagnosed with non-small cell (NSCLC) or small cell lung cancer (SCLC) who were recorded in the NLCA between 1st January 2004 and 31^st^ December 2011 inclusive. Those who did not have a date of death recorded on the final date that ONS records were updated (March 2013–14 months after the last patient entry into the dataset) were presumed to still be alive and therefore excluded from further analysis.

### Covariate descriptions

Comorbidity was classified according to Charlson score, which was derived to predict the ten-year mortality for patients with a list of 19 co-morbid conditions. [9]. Clinical coders enter a primary diagnosis or diagnoses and also a list of co-morbidities for each patient episode in HES. ICD-10 codes from linked-HES data were used to identify relevant diagnoses which contribute to the Charlson score. Each condition is assigned a score of 1, 2, 3, or 6, depending on the risk of dying associated with each one and summed to produce a single value for each patient. As all patients had lung cancer this was not included in the ‘any tumour’ section. Patients were then categorised into five groups based on their Charlson index score (0, 1, 2, 3 and ≥ 4). Townsend Index of deprivation was used as a measure of socioeconomic status. This is a post-code derived variable which uses 4 markers of social deprivation derived from census records to categorise patients into quintiles; where 5 is the most deprived and 1 the most affluent grouping. [10] Pre-treatment records in NLCA were used for stage (grouped as IA/IB, IIA/IIB, IIIA, IIIB, IV, SCLC limited or SCLC extensive) according to the 7^th^ edition of UICC TNM, with post-treatment stage used if pre-treatment records were missing. We used pre-treatment recording of histology and again post-treatment records were used if this was missing. The NLCA uses the Eastern Cooperative Oncology Group (ECOG) definition of performance status (PS). This is a subjective marker which is used to try to quantify general well-being and ability to carry out activities of daily life and ranges from 0–5, where 0 is fully active, able to carry on all pre-disease performance without restriction and 4 is completely disabled; cannot carry on any self-care; totally confined to bed or chair (5 is dead).” [11] We used age at the time of diagnosis to categorise patients into eight groups (<55, 55–59, 60–64, 65–69, 70–74, 75–79, 80–84 and ≥85 years). NLCA records were used to identify source of referral. This refers to the source of referral to the lung cancer team and comprises two emergency routes (following an emergency admission or following an accident and emergency (A&E) attendance) and seven semi-elective routes which include GP referral and referral from another secondary care provider or allied health professional.

ONS records were used to characterise place of death as home, acute hospital, residential/nursing home, hospice, community/rehabilitation hospital or elsewhere. Cause of death was defined by ONS using the criteria set out in the 10th revision of the “International classification of diseases” (ICD-10) to determine a single underlying cause of death.[12] England was divided into 28 cancer networks at the time this data was collected so we used these to assess geographical variation in place of death. We calculated the proportion of patients dying in each location and stratified this by cancer network. We also aimed to look at changes in place of death over the duration of the NLCA. We created four groups based on year of death (2004–2006, 2007–2008, 2009–2010 and 2011–2013) and looked at the proportion of patients dying in each place, as described previously, for each year grouping. Work from the Routes From Diagnosis project showed that 50% of lung cancer patients died within 6 months of diagnosis.[13] To try to identify if there was a difference in place of death related to time from diagnosis we identified those who died within 6 months of diagnosis and compared the proportion dying in each location with those who died more than 6 months after diagnosis.

### Statistical methods

All statistical analyses were conducted using Stata MP V.12 (StataCorp, Texas, USA). In order to compare the factors influencing death in the least preferred location (acute hospital) with home, which is frequently reported to be the desired place of death, we excluded those who died in other locations from this part of the analysis. Given the median age of diagnosis for lung cancer is 72 years in the UK we used this as our reference category rather than the youngest age grouping for analysis. Logistic regression was used to calculate odds ratios (OR) for death within an acute hospital bed within each of the variables described above. We looked at each variable individually initially, then included those which showed an association in the univariate model (p<0.05) in the multivariate analysis. Likelihood ratio testing (LRT) was used for all tests of significance. We conducted a sensitivity analysis, excluding those with missing data, to ensure that our findings were robust. There was minimal change in odds ratios and 95% confidence intervals, so the results of the original multivariate analysis including all patients are presented in the results section.

The Healthcare Quality Improvement Partnership (HQIP) approved the use of the National Lung Cancer Audit for this work. This study does not involve identifiable human participants- an anonymized dataset was obtained via the HSCIC (under the terms of a data sharing agreement (reference RU943-R1)) who hold data extracted and anonymised specifically for research use. No study authors had any role in data collection and no personal patient information was provided in the data which was obtained by the authors. Analysis of these data were reviewed and approved by the University of Nottingham research ethics committee in December 2012 (reference B15112012LT CHS EPH).

## Results

### Patient population and data source

Records were identified for 178,178 patients in the NLCA. Of these 24150 were excluded as they were still alive at the time ONS records were last accessed for dates of death and a further 8702 patients with carcinoid or mesothelioma histological subtypes, 1417 with dates of diagnosis after their date of death and 282 with missing information on place of death were also excluded. This left a final cohort of 143,627 patients.

### Demographic features

Overall 58% were male, with a median age at diagnosis of 73 years (interquartile range (IQR) 65–79 years). The majority had NSCLC (88%) and most patients had advanced disease at presentation; 39% had stage IV NSCLC, 12% stage IIIB, with stages IA to IIA NSCLC comprising 18% collectively. A large proportion of patients were from the most deprived socioeconomic groups (40% from Townsend quintiles 4 and 5), with a median Charlson co-morbidity score of 1 (IQR 0–3). Median survival from the time of diagnosis was 145 days (IQR 47–346 days).

### Place of death

Forty per cent of patients (57678) died in acute hospital beds, 29% (41957) in their own home, 17% (24108) in hospice beds, 8% (12099) died in residential or nursing homes, 4% (5517) in community or rehabilitation hospitals and 2% (2268) “elsewhere”; which contained those dying within prisons and mental health institutions. A summary of the demographics of those dying in each location is provided in Table 1. Those dying in residential or nursing homes were older than the other localities and had worse WHO performance status at diagnosis (33% PS 3 or 4). Proportionally more of the younger age groups died within hospices. Those from the most affluent socioeconomic groups (Townsend quintiles 1 and 2) were most likely to die either in a hospice or at home.

**Table 1:** Demographics of patients stratified by place of death.

		Home		Acute hospital		Residential/ nursing home		Hospice		Community/ rehab hospital		Elsewhere		Total	
		N = 41957		N = 57678		N = 12099		N = 24108		N = 5517		N = 2268		N = 143627	
		n	%	n	%	n	%	n	%	n	%	n	%	n	%
**Sex**	**Female**	**17794**	**42.4**	**22820**	**39.6**	**5926**	**49**	**10851**	**45**	**2341**	**42.4**	**1208**	**53.3**	**60940**	**42.4**
	**Male**	**24163**	**57.6**	**34858**	**60.4**	**6173**	**51**	**13257**	**55**	**3176**	**57.6**	**1060**	**46.7**	**82.687**	**57.6**
**Age group**	**<55**	**2593**	**6.2**	**3562**	**6.2**	**151**	**1.3**	**2022**	**8.4**	**199**	**3.6**	**173**	**7.6**	**8700**	**6.1**
	**55–59**	**3161**	**7.5**	**4173**	**7.2**	**300**	**2.5**	**2103**	**8.7**	**264**	**4.8**	**162**	**7.1**	**10163**	**7.1**
	**60–64**	**5276**	**12.6**	**6895**	**12**	**621**	**5.1**	**3292**	**13.7**	**470**	**8.5**	**239**	**10.5**	**16793**	**11.7**
	**65–69**	**6570**	**15.7**	**8780**	**15.2**	**1013**	**8.4**	**3802**	**15.8**	**701**	**12.7**	**333**	**14.7**	**21199**	**14.8**
	**70–74**	**7609**	**18.1**	**10347**	**17.9**	**1576**	**13**	**4144**	**17.2**	**903**	**16.4**	**357**	**15.7**	**24936**	**17.4**
	**75–79**	**7791**	**18.6**	**10401**	**18**	**2369**	**19.6**	**4048**	**16.8**	**1111**	**20.1**	**394**	**17.4**	**26114**	**18.2**
	**80–84**	**5732**	**13.7**	**8066**	**14**	**2830**	**23.4**	**2994**	**12.4**	**1096**	**19.9**	**360**	**15.9**	**21078**	**14.7**
	**85+**	**3225**	**7.7**	**5454**	**9.5**	**3239**	**26.8**	**1703**	**7.1**	**773**	**14**	**250**	**11**	**14644**	**10.2**
**Performance status**	**0**	**5513**	**13.1**	**6874**	**11.9**	**820**	**6.8**	**3264**	**13.5**	**547**	**9.9**	**255**	**11.2**	**17273**	**12**
	**1**	**10513**	**25.1**	**13229**	**22.9**	**1942**	**16.1**	**6187**	**25.7**	**1180**	**21.4**	**514**	**22.7**	**33565**	**23.4**
	**2**	**7512**	**17.9**	**9446**	**16.4**	**2099**	**17.4**	**4084**	**16.9**	**899**	**16.3**	**432**	**19.1**	**24472**	**17**
	**3**	**6341**	**15.1**	**8625**	**15**	**2910**	**24.1**	**3148**	**13.1**	**910**	**16.5**	**372**	**16.4**	**22306**	**15.5**
	**4**	**1451**	**3.5**	**3657**	**6.3**	**1128**	**9.3**	**794**	**3.3**	**278**	**5**	**81**	**3.6**	**7389**	**5.1**
	**Missing**	**10627**	**25.3**	**15847**	**27.5**	**3200**	**26.5**	**6631**	**27.5**	**1703**	**30.9**	**614**	**27.1**	**38622**	**26.9**
**Charlson score**	**0**	**16627**	**39.6**	**21170**	**37.6**	**4158**	**34.4**	**10121**	**42**	**2164**	**39.2**	**965**	**42.6**	**55745**	**38.8**
	**1**	**8553**	**20.4**	**12162**	**21.1**	**2594**	**21.4**	**4623**	**19.2**	**1120**	**20.3**	**466**	**20.6**	**29518**	**20.6**
	**2**	**5039**	**12**	**7124**	**12.4**	**1641**	**13.6**	**2674**	**11.1**	**631**	**11.4**	**253**	**11.2**	**17362**	**12.1**
	**3**	**2605**	**6.2**	**3944**	**6.8**	**1026**	**8.5**	**1358**	**5.6**	**368**	**6.7**	**120**	**5.3**	**9421**	**6.6**
	**≥4**	**9133**	**21.8**	**12738**	**22.1**	**2680**	**22.2**	**5332**	**22.1**	**1234**	**22.4**	**464**	**20.5**	**31581**	**22**
**Townsend quintile**	**1**	**6000**	**14.3**	**7029**	**12.2**	**1766**	**14.6**	**3456**	**14.3**	**748**	**13.6**	**307**	**13.5**	**19306**	**13.4**
	**2**	**7262**	**17.3**	**8531**	**14.8**	**2111**	**17.5**	**4129**	**17.1**	**1096**	**19.9**	**376**	**16.6**	**23505**	**16.4**
	**3**	**7277**	**17.3**	**9641**	**16.7**	**2452**	**20.3**	**4282**	**17.8**	**1151**	**20.9**	**359**	**15.8**	**25162**	**17.5**
	**4**	**8002**	**20.6**	**11175**	**19.4**	**2400**	**19.8**	**4362**	**18.1**	**1031**	**18.7**	**459**	**20.2**	**27429**	**19.1**
	**5**	**8647**	**20.6**	**13358**	**23.2**	**2046**	**16.9**	**4807**	**19.9**	**606**	**11**	**516**	**22.8**	**29980**	**20.9**
	**Missing**	**4769**	**11.4**	**7944**	**13.8**	**1324**	**10.9**	**3072**	**12.7**	**885**	**16**	**251**	**11.1**	**18245**	**12.7**
**Histology**	**Adenocarcinoma**	**6790**	**16.2**	**9248**	**16**	**1348**	**11.1**	**4506**	**18.7**	**748**	**13.6**	**377**	**16.6**	**23017**	**16**
	**Squamous cell**	**7313**	**17.4**	**9433**	**16.4**	**1615**	**13.4**	**3772**	**15.7**	**837**	**15.2**	**380**	**16.8**	**23350**	**16.3**
	**NSCLC NOS**	**7746**	**18.5**	**10155**	**17.6**	**1549**	**12.8**	**4653**	**19.3**	**993**	**18**	**383**	**16.9**	**25479**	**17.7**
	**SCLC**	**5384**	**12.8**	**6954**	**12.1**	**928**	**7.7**	**3037**	**12.6**	**604**	**11**	**282**	**12.4**	**17189**	**12**
	**Other**	**1721**	**4.1**	**2205**	**3.8**	**444**	**3.7**	**1055**	**4.4**	**193**	**3.5**	**89**	**3.9**	**5707**	**4**
	**Missing**	**13003**	**31**	**19683**	**34.1**	**6215**	**51.4**	**7085**	**29.4**	**2412**	**38.8**	**757**	**33.4**	**48885**	**34**
**Stage**	**IA/ IB**	**2325**	**5.5**	**3904**	**6.8**	**990**	**8.2**	**1140**	**4.7**	**288**	**5.2**	**110**	**4.9**	**8757**	**6.1**
	**IIA/ IIB**	**1589**	**3.8**	**2262**	**3.9**	**587**	**4.9**	**820**	**3.4**	**175**	**3.2**	**83**	**3.7**	**5516**	**3.8**
	**IIIA**	**3351**	**8**	**4156**	**7.2**	**1020**	**8.4**	**1833**	**7.6**	**370**	**6.7**	**160**	**7.1**	**10890**	**7.6**
	**IIIB**	**5356**	**12.8**	**7013**	**12.2**	**1305**	**10.8**	**2935**	**12.2**	**654**	**11.9**	**290**	**12.8**	**17553**	**12.2**
	**IV**	**16773**	**40**	**21605**	**37.5**	**4414**	**36.5**	**9928**	**41.2**	**2036**	**36.9**	**920**	**40.6**	**55676**	**38.8**
	**SCLC Limited**	**448**	**1.1**	**536**	**0.9**	**75**	**0.6**	**225**	**0.9**	**48**	**0.9**	**14**	**0.6**	**1346**	**0.9**
	**SCLC Extensive**	**1047**	**2.5**	**1502**	**2.6**	**152**	**1.3**	**635**	**2.6**	**149**	**2.7**	**58**	**2.6**	**3543**	**2.5**
	**Missing**	**11068**	**26.4**	**16700**	**29**	**3556**	**29.4**	**6592**	**27.3**	**1797**	**32.6**	**633**	**27.9**	**40346**	**28.1**
**Source of referral**	**Emergency admission**	**5012**	**12**	**9096**	**15.8**	**2243**	**18.5**	**2708**	**11.2**	**951**	**17.2**	**308**	**13.6**	**20318**	**14.2**
	**GP referral**	**21255**	**50.7**	**25453**	**44.1**	**4809**	**39.8**	**11651**	**48.3**	**2607**	**47.3**	**1018**	**44.9**	**66793**	**46.5**
	**Consultant referral**	**8092**	**19.3**	**11287**	**19.6**	**2495**	**20.6**	**4719**	**19.6**	**928**	**16.8**	**456**	**20.1**	**27977**	**19.5**
	**A&E**	**2514**	**6**	**4485**	**7.8**	**1077**	**8.9**	**1534**	**604**	**353**	**6.4**	**183**	**8.1**	**10146**	**7.1**
	**Other**	**2787**	**6.6**	**4032**	**7**	**814**	**6.7**	**1807**	**7.5**	**283**	**5.1**	**186**	**8.2**	**9903**	**6.9**
	**Unknown**	**2297**	**5.5**	**3325**	**5.8**	**661**	**5.5**	**1695**	**7**	**395**	**7.2**	**117**	**5.2**	**8490**	**5.9**
**Death within 6 months of diagnosis**	**0–6 months**	**21574**	**51.4**	**35504**	**61.6**	**6398**	**52.9**	**12105**	**50.2**	**3281**	**59.5**	**1367**	**60.3**	**80229**	**55.9**
	**> 6 months**	**20383**	**48.6**	**22174**	**38.4**	**5701**	**47.1**	**12003**	**49.8**	**2236**	**40.5**	**901**	**39.7**	**63398**	**44.1**

### Multivariable analysis

In order to compare the odds of death in an acute hospital with home we excluded the 43992 patients who died in the other locations, which left us with a cohort of 99635 patients for the univariable and multivariable analyses. The results are presented in Table 2. Males were more likely than females to die in acute hospital beds compared with home (adjusted odds ratio (OR) 1.14; 95% confidence interval (95% CI) 1.12–1.17). Those in the oldest age bracket (85 years and older) were at increased odds of dying in acute hospital beds (adjusted OR 1.16; 95% CI 1.08–1.24) compared with all of the younger age groups. There was no real difference between the other age brackets and likelihood of death within an acute hospital bed. Patients who presented at WHO performance status 4 (bed bound) were more likely to die in acute hospitals compared with those who were fitter (adjusted OR 1.73; 95% CI 1.61–1.87 for WHO PS 4 compared to WHO PS 0). Again there was only an impact on likelihood of dying with an acute hospital in the least fit group (WHO PS 4), with no difference between the other classes in the multivariable analysis. There were increasing odds of death within acute hospitals with increasing levels of socioeconomic deprivation (adjusted OR 1.31; 95% CI 1.25–1.37) for Townsend quintile 5 versus quintile 1). Compared with diagnosis via a general practitioner (GP) referral via all other routes was associated with an increased chance of dying within an acute hospital bed. This was most marked for the emergency admission route (adjusted OR 1.45; 95% CI 1.39–1.51) and in those diagnosed following attendance at accident and emergency (A&E) departments (adjusted OR 1.43; 95% CI 1.35–1.51). Those with highest stage disease (stage IV) at diagnosis were less likely to die in acute hospital beds when compared with those with earlier stage disease at presentation (adjusted OR 0.74; 95% CI 0.70–0.79). In fact those with stage IA and IB NSCLC were more likely to die in acute hospital beds than all other stages of NSCLC and also than those with limited and extensive stage SCLC.

**Table 2:** Results of univariate and multivariate analysis comparing death in an acute hospital versus death at home.

		Univariate analysis			Multivariate analysis		
		Death in acute hospital		pvalue	Death in acute hospital		pvalue
		OR	95% CI	(LRT)	OR	95% CI	(LRT)
**Sex**	**Female**	1		p<0.01	1		p<0.01
	**Male**	1.28	1.26–1.31		1.14	1.12–1.17	
**Age group**	**<55**	0.96	0.90–1.03		0.97	0.91–1.04	
	**55–59**	0.95	0.89–1.01		0.97	0.91–1.03	
	**60–64**	0.97	0.92–1.03		0.98	0.92–1.02	
	**65–69**	0.99	0.93–1.05		0.98	0.93–1.04	
	**70–74**	1		p<0.01	1		p<0.01
	**75–79**	0.97	0.92–1.03		0.95	0.90–1.01	
	**80–84**	1.02	0.96–1.09		0.99	0.93–1.06	
	**85+**	1.23	1.15–1.32		1.16	1.08–1.24	
**Performance status**	**0**	1		p<0.01	1		p<0.01
	**1**	1.01	0.97–1.05		0.99	0.95–1.03	
	**2**	1.01	0.96–1.06		0.95	0.90–0.99	
	**3**	1.09	1.04–1.14		0.97	0.93–1.03	
	**4**	2.02	1.88–2.17		1.73	1.61–1.87	
	**Missing**	1.2	1.15–1.25		1.08	1.03–1.13	
**Charlson score**	**0**	1		p<0.01	1		p = 0.251
	**1**	1.09	1.05–1.13		1.04	1.00–1.08	
	**2**	1.08	1.04–1.13		1.02	0.98–1.06	
	**3**	1.16	1.10–1.22		1.06	1.01–1.12	
	**≥4**	1.07	1.03–1.10		0.99	0.96–1.03	
**Townsend quintile**	**1**	1		p<0.01	1		p<0.01
	**2**	1	0.96–1.05		1.01	0.96–1.06	
	**3**	1.13	1.08–1.18		1.13	1.08–1.18	
	**4**	1.19	1.14–1.25		1.19	1.13–1.24	
	**5**	1.32	1.26–1.38		1.31	1.25–1.37	
	**Missing**	1.42	1.35–1.49		1.38	1.31–1.46	
**Histology**	**Adenocarcinoma**	1		p<0.01	1		p = 0.026
	**Squamous cell**	0.95	0.91–0.99		0.92	0.88–0.97	
	**NSCLC NOS**	0.96	0.92–1.00		0.97	0.93–1.01	
	**SCLC**	0.95	0.90–0.99		0.92	0.88–0.98	
	**Other**	0.94	0.88–1.01		0.92	0.86–0.99	
	**Missing**	1.11	1.07–1.15		0.97	0.94–1.01	
**Stage**	**IA/ IB**	1		p<0.01	1		p<0.01
	**IIA/ IIB**	0.85	0.78–0.92		0.86	0.79–0.94	
	**IIIA**	0.74	0.69–0.79		0.76	0.71–0.82	
	**IIIB**	0.78	0.73–0.83		0.79	0.74–0.84	
	**IV**	0.77	0.73–0.81		0.74	0.70–0.79	
	**SCLC Limited**	0.71	0.62–0.82		0.76	0.66–0.87	
	**SCLC Extensive**	0.85	0.78–0.94		0.86	0.77–0.95	
	**Missing**	0.9	0.85–0.95		0.84	0.79–0.89	
**Source of referral**	**Emergency admission**	1.52	1.46–1.58		1.45	1.39–1.51	
	**GP referral**	1		p<0.01	1		p<0.01
	**Consultant referral**	1.16	1.13–1.20		1.13	1.09–1.17	
	**A&E**	1.49	1.41–1.57		1.43	1.35–1.51	
	**Other**	1.21	1.15–1.27		1.16	1.10–1.23	
	**Unknown**	1.21	1.14–1.28		1.16	1.09–1.23	

LRT- likelihood ratio test

### Geographical variation in place of death

**Fig 1:**
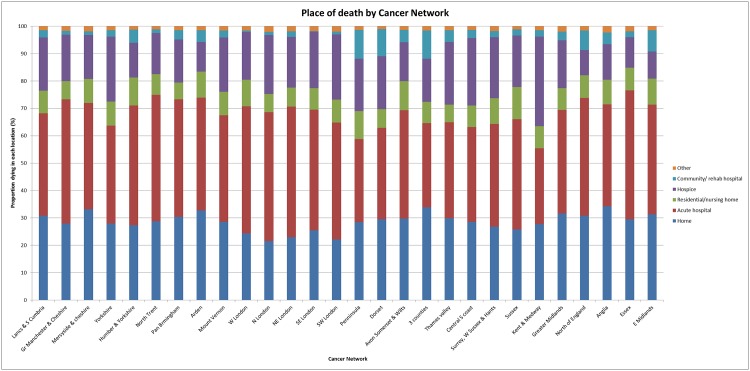
Place of death stratified by cancer Network.

There was evidence of marked variation in place of death when we stratified patients by each of the 28 cancer networks (Fig 1). These regional inequalities were most marked in the proportion of patients dying within hospices, with only 9% in the lowest network dying within this setting compared with 33% in the highest. There was a 20% difference in the proportion dying in acute hospital beds between the lowest and highest cancer network (28% to 48%). There was similar variation in the proportion of patients dying in their own home (22% versus 34% in the highest and lowest networks respectively).

### Changes over time

**Fig 2:**
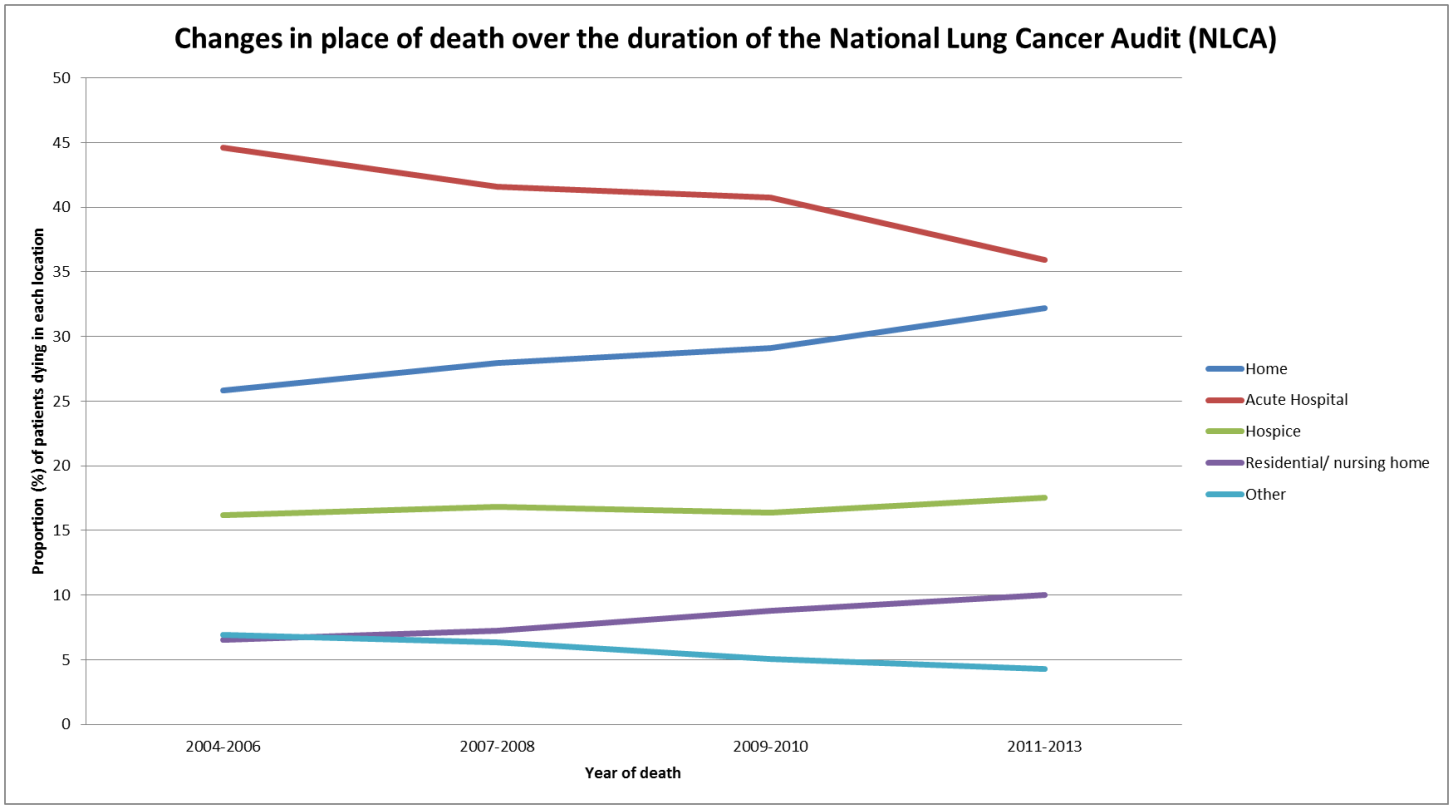
Changes in place of death between 2004 and 2013.

Changes in place of death between 2004 and 2013 were also assessed (Fig 2), showing a decrease in the proportion of patients dying in acute hospital beds from 45% in 2004–2006 to 35% in 2011–2013. There has been a corresponding increase in the proportion dying at home (26% in 2004–2006 compared to 32% in 2011–2013) but only a very small increase (from 16% to 18%) in the proportion dying within hospice beds. An increase in those dying within residential and nursing home beds has been mirrored by a reduction in the number of community hospital beds and corresponding decrease in the proportion dying within that setting.

### Time from diagnosis to death

Finally we looked at place of death in those who died in the 6 months following the lung cancer diagnosis and compared this with those who survived for more than 6 months. These results are summarised in Table 3. Fifty six per cent of our patients died within the 0–6 month time frame. Proportionally more of these patients died in acute hospital beds (44% compared to 35% of those who lived for over 6 months). Patients who survived for over 6 months after their diagnosis were more likely to die at home (32% versus 27%) or in a hospice setting (19% versus 15%) than those who died in the first 6 month window. Those who died within the initial 6 months after diagnosis were older (median age at diagnosis of 74 years (IQR 66–80) compared with median age at diagnosis of 71 years (IQR 63–78) in the group who lived for longer than 6 months). There were similar proportions of males and females between the 2 groups (58% males in the group who died within 6 months of diagnosis versus 56% in the group who survived for longer). There was a marked difference in the distribution of performance status when comparing the 2 groups; 50% of those in the group who survived more than 6 months were deemed to be WHO PS 0 or 1 at diagnosis compared with only 23% of those in the group who died within 0–6 month time frame. There were proportionally more patients who were felt to be WHO PS 3 or 4 in the cohort who died within 6 months (30% versus 8%). Seventeen per cent of those in the group who lived for longer than 6 months presented with lung cancer that was stage IA-IIB compared with only 5% in the group who died within 6 months, whilst 48% of the group who died within 6 months had stage IV disease at presentation versus 27% in the group who survived for longer. The group who died within 6 months were also proportionally more likely to be diagnosed via an emergency admission route (28% versus 12%), with only 40% referred from their GP compared with 55% in the group who lived for longer than 6 months.

**Table 3:** Demographic features of patients comparing those who died within 0–6 months following diagnosis and > 6 months.

		Death within 0–6 months of diagnosis		Death > 6 months after diagnosis	
		N = 80229	%	N = 63398	%
**Place of death**	**Home**	21574	27	20383	32
	**Acute hospital**	35504	44	22174	35
	**Residential/ nursing home**	6398	8	5701	9
	**Hospice**	12105	15	12003	19
	**Community/ rehab hospital**	3281	4	2236	4
	**Elsewhere**	1367	2	901	1
**Sex**	**Female**	33333	42	27607	44
	**Male**	46896	58	35791	56
**Median age at diagnosis**	**Years (IQR)**	74 (66–80)		71 (63–78)	
**Performance status**	**0**	5110	6	12163	19
	**1**	13858	17	19707	31
	**2**	15054	19	9418	15
	**3**	17578	22	4728	7
	**4**	6731	8	658	1
	**Missing**	21898	27	16724	26
**Stage**	**IA/ IB**	2061	3	6807	11
	**IIA/ IIB**	1713	2	3803	6
	**IIIA**	4030	5	6860	11
	**IIIB**	8439	11	9114	14
	**IV**	38540	48	17136	27
	**SCLC Limited**	378	0.5	968	2
	**SCLC Extensive**	2148	3	1395	2
	**Missing**	22920	29	17315	27
**Source of referral**	**Emergency admission**	15269	19	5049	8
	**GP referral**	31763	40	35030	55
	**Consultant referral**	15760	20	12217	19
	**A&E**	7476	9	2670	4
	**Other**	5260	7	4643	7
	**Unknown**	4701	6	3789	6

### Cause of death

Cause of death was recorded as lung cancer in 86% (123147) of patients. Death was attributed to other malignancies in 6% (8996) of cases, chronic obstructive pulmonary disease (COPD) in 1.4% (2021) and ischaemic heart disease (IHD) in 1.5% (2172), with a variety of other conditions comprising the remaining 5%.

## Discussion

Our results show that, although there has been a 10% reduction in the proportion of patients with lung cancer dying in acute hospital beds between 2004 and 2013, 35% still die within that setting. Old age (≥ 85 years), male sex, socioeconomic deprivation, WHO performance status 4 at diagnosis and emergency route to diagnosis are all strongly and independently associated with increased likelihood of death within acute hospitals, although death in this location is less likely with more advanced stage disease at presentation. In additional there is evidence of geographical variation in place of death, particularly with regard to provision of hospice services. Given the often advanced stage of disease at presentation and poor prognosis it is unsurprising that the majority of patients with a diagnosis of lung cancer are recorded as having died of the underlying disease.

### Strengths and limitations

This is one of the first studies to look in detail at both place and cause of death in lung cancer patients specifically and the large number of patients and representative nature of the population is a particular strength. The NLCA has previously been validated as a reliable source of the UK lung cancer data and currently has in excess of 90% case ascertainment. [14] Although data completeness is improving in the NLCA, missing data are still a challenge. A sensitivity analysis conducted excluding those with missing data fields did not change any of the results substantially suggesting that the missing data did not affect the validity of our findings. The population in NLCA is predominantly white which does make extrapolation of these results to other ethnic groupings difficult. In addition work by Gao et al [6] has suggested that marital status may be relevant to place of death; with those who are single, widowed or divorced being more likely to die in the hospital setting. These data are not routinely recorded in our datasets so there may be confounding due to inability to adjust for this. We were also unable to take into account patient preference in place of death or assess whether this might change when faced with worsening symptoms in the terminal phase of the illness. However, although we accept that not all patients will choose to die at home existing evidence suggests that this is the preference for most. [1]

### Other work in the literature

Gao and colleagues used ONS data to look at changes in place of death for all cancer deaths in England between 1993 and 2010.[6] They also looked at some features associated with death at home or in a hospice. Although there was a decrease in the overall proportion of cancer patients dying in hospital over this time frame there was little change in the lung cancer group. Similar to our study they found that male sex, increasing age, social deprivation and in addition marital status were independently associated with increased likelihood of dying in acute hospitals. A further piece of work by Gomes et al [15] looked at changes in place of death between 2004 and 2010 for both cancer and non-cancer patients and showed that there has been a small rise in the proportion of patients overall dying at home over this time period; with 27% of all cancer patients dying in their own home in 2010. This is very similar to the 29% we observed in the lung cancer population for the years 2009–2010. In addition Davies et al [16] assessed changes in place of death for cancer patients in the Thames Cancer Registry over a 17 year period (1985–2002), showing a decrease in the proportion of patients dying within acute hospital beds from 67% to 47% by 2002. They also showed that those who were older (> 75 years) were more likely to die in acute hospital beds. A randomised controlled trial conducted by Brumley et al [17] assigned patients with a terminal prognosis to usual care or in-home palliative care, which comprised not only pain and symptom relief, but a variety of educational, medical and social support services. They looked at visits to the emergency department (ED) and also place of death in both groups and showed that the group receiving palliative care support had fewer visits to the ED and also were 20% more likely to die at home compared to the usual care arm. This highlights the key role that early and effective palliative care has to play in ensuring that wherever possible patients are able to achieve their preferred place of death. Studies consistently show that patients with lung cancer are more likely to die in acute hospital beds than patients with many other solid organ tumours and there is a suggestion that this is due to late discussions about end-of-life care [7] in combination with a rapidly progressive disease. A study looking at hospitalisations towards the end of life specifically in poor-prognosis cancer types in the United States showed that high-intensity inpatient care (including administration of chemotherapy and admission to Intensive care units) was delivered close to death in many patients, which may prevent better supportive care being delivered and end-of-life decisions being made in a timely manner.[18] There is evidence that patients who discuss end of life care preferences are more likely to die within home or hospice settings however we did not have the data available to know what extent these factors contributed to death within acute hospital settings in our cohort.[19,20] It may be that those patients who survived for more than 6 months after diagnosis had more time to plan and discuss preferences for end of life care than those who died earlier in the disease process possibly explaining in part the smaller proportion of patients in this group who died in acute hospital beds when compared with those who died in the 6 months immediately following their diagnosis. A variety of patient and tumour features also contribute to mortality within the initial 6 months following diagnosis which may make discharge from acute hospital beds more difficult to facilitate. Work from the National Cancer Intelligence Network (NCIN) “Routes to Diagnosis” project has shown that lung cancer patients who present via the emergency route have a much lower 1 year survival [21] which explains why proportionally more patients in the group who died within 0–6 months were diagnosed via an emergency admission route. In addition performance status has been previously shown to be an independent predictor for overall survival in lung cancer, with the differences in performance status marked between those who died within 6 months and those who lived for longer in our cohort.[22]

Work performed in South East England has suggested that those from ethnic minority groups were more likely to die in hospital beds and less likely to access hospice services than white patients, which suggests there may be an additional need to look in more detail at the ethnicity of patients and the part it has to play in place of death.[23] The wide geographic variations in place of death are likely to represent service availability and accessibility. A study by Gatrell et al [24] looked at variations in geographic access to hospices in England and Wales and showed that there are inequities in provision of beds between regions, with the lowest numbers of beds per 1000 cancer deaths in the North East and East Midlands; which corresponds to the two cancer Networks with the lowest proportion of patients dying within hospice beds in our study (North of England and East Midlands). Furthermore a piece of work from Sleeman et al [25] suggested that those from a more affluent background were more likely to die in a hospice setting than those from the most deprived socio-economic grouping, suggesting that inequities in access are not only geographic.

### Interpretation of the results

The fact that those with higher stage disease at presentation are less likely to die within an acute hospital setting was an unexpected finding, however it may indicate that we are better at offering early palliative care to patients with non-curative disease to try to ensure that they achieve their preference in place of death compared with those who undergo surgery or aggressive systemic anti-cancer treatment; where the focus is on management with curative intent rather than end of life care. Those with higher stage disease would also therefore be less likely to suffer morbidity and mortality related to treatment side effects which may necessitate hospitalisation in the latter days of life. It may also be the case that recognition that the patient has reached the terminal phase of the disease, with a shift in the treatment paradigm from curative to palliative, may occur too late to allow the preference in place of death to be met. There is no study which has specifically addressed this question however. It is difficult to suggest what the optimal proportion of patients dying in acute hospital settings should be as many patients develop distressing symptoms in the latter days of life which necessitate hospitalisation. Only 28% of patients in the lowest cancer network died in acute hospital beds (due principally to a large number of in-hospice deaths in this region) so it is not unreasonable to suggest that this could be the gold standard given optimal service provision and resource allocation, although we recognise that there are differing social and patient demographic features which also may influence this between regions. Another option may be to optimise palliative services for these patients on site at acute hospitals or provide a more integrated community and acute hospital service than many regions currently have to facilitate discharge to a more appropriate setting.

### Clinical relevance and conclusions

A substantial proportion of patients with lung cancer die in acute hospital beds and this is more likely in the most elderly (≥85 years), males, those who are socially deprived, those diagnosed via an emergency admission route and in those with the worst performance status (WHO PS 4). There is marked geographical variation, suggesting a need to improve end-of-life planning in those at greatest risk, and to review the allocation of resources to provide more hospice beds, enhanced community support and ensure equal access. Increasing the proportion of patients dying in the community would undoubtedly offer cost savings in addition to psychological benefits.
